# Chromosomal Minimal Critical Regions in Therapy-Related Leukemia Appear Different from Those of *De Novo* Leukemia by High-Resolution aCGH

**DOI:** 10.1371/journal.pone.0016623

**Published:** 2011-02-14

**Authors:** Nathalie Itzhar, Philippe Dessen, Saloua Toujani, Nathalie Auger, Claude Preudhomme, Catherine Richon, Vladimir Lazar, Véronique Saada, Anelyse Bennaceur, Jean Henri Bourhis, Stéphane de Botton, Alain Bernheim

**Affiliations:** 1 Institut de la Santé et de la Reherche Médicale U985, Génétique des tumeurs, Institut Gustave Roussy, Villejuif, France; 2 Université Paris XI, Paris Sud, Orsay, France; 3 Molecular Pathology, Villejuif, France; 4 Institut Gustave Roussy, Functional Genomics Unit, Institut Gustave Roussy, Villejuif, France; 5 Department of Hematology, Centre de Biologie-Pathologie, Centre Hospitalier Régional Universitaire de Lille, Lille, France; 6 Department of Hematology, Institut Gustave Roussy, Villejuif, France; Innsbruck Medical University, Austria

## Abstract

Therapy-related acute leukemia (t-AML), is a severe complication of cytotoxic therapy used for primary cancer treatment. The outcome of these patients is poor, compared to people who develop *de novo* acute leukemia (p-AML). Cytogenetic abnormalities in t-AML are similar to those found in p-AML but present more frequent unfavorable karyotypes depending on the inducting agent. Losses of chromosome 5 or 7 are observed after alkylating agents while balanced translocations are found after topoisomerase II inhibitors. This study compared t-AML to p-AML using high resolution array CGH in order to find copy number abnormalities (CNA) at a higher resolution than conventional cytogenetics. More CNAs were observed in 30 t-AML than in 36 p-AML: 104 CNAs were observed with 63 losses and 41 gains (mean number 3.46 per case) in t-AML, while in p-AML, 69 CNAs were observed with 32 losses and 37 gains (mean number of 1.9 per case). In primary leukemia with a previously “normal” karyotype, 18% exhibited a previously undetected CNA, whereas in the (few) t-AML with a normal karyotype, the rate was 50%. Several minimal critical regions (MCRs) were found in t-AML and p-AML. No common MCRs were found in the two groups. In t-AML a 40kb deleted MCR pointed to *RUNX1* on 21q22, a gene coding for a transcription factor implicated in frequent rearrangements in leukemia and in familial thrombocytopenia. In *de novo* AML, a 1Mb MCR harboring *ERG* and *ETS2* was observed from patients with complex aCGH profiles. High resolution cytogenomics obtained by aCGH and similar techniques already published allowed us to characterize numerous non random chromosome abnormalities. This work supports the hypothesis that they can be classified into several categories: abnormalities common to all AML; those more frequently found in t-AML and those specifically found in p-AML.

## Introduction

Therapy-related myeloblastic leukemia, including therapy-related myelodysplasia, (t-AML), constitutes approximately 10% of AML and has several characteristic features [1], [2]. As the incidence of cancers increases, so does that of t-AML. Nowadays, thanks to treatment intensification, the cure rate of primary neoplasia has increased but these very treatments are also implicated in severe therapy-related hematological consequences.

Factors associated with the development of t-AML include prior cytotoxic therapy. Two different types of t-AML are to be distinguished. The first one is due to prior therapy with alkylating agents (AA) or radiotherapy [1], [2], [3], [4]. It occurs generally after a latency period of 5 to 7 years. This kind of t-AML is often preceded by a preleukemic period of myelodysplasia (t-MDS). Approximately 90% of the patients with AA-related t-AML exhibit clonal chromosomal aberrations including monosomy or deletions on chromosome 5 and/or 7, or complex aberrations involving chromosome 3, 12, 17 and 21. Drugs that target topoisomerase II (ATII), such as etoposide and anthracyclines may induce the second type of t-AML [1], [2], [3], [4]. It occurs in a median of 2 years and is not preceded by MDS. Cytogenetic analysis shows a high frequency of rearrangements of chromosome band 11q23 but also recurrent balanced rearrangements t(8;21), t(15;17) and inv(16) [1], [2].

The prognosis is poor in t-AML, excepted in case of t(8;21), t(15;17) and inv(16) which follow the same course as p-AML and the karyotype is more frequently modified with at least 2 abnormalities or more.

p-AML are also heterogeneous entities, classified according to bone marrow cell morphology and karyotype with the recent addition of several gene mutations. AML was among the first diseases to be treated and monitored according to somatic acquired chromosomal abnormalities, including the first successful targeted treatment against a pathological gene product in AML3.

As t-AML appear to have a particular leukemogenesis pathway [3], [4], the aims of this work were:

1) to analyze t-AML using high resolution array CGH in order to find genome region or even gene-specific copy number abnormalities (CNA) at a higher resolution than conventional cytogenetics; 2) to delimitate minimal critical regions (MCR) in order to identify potential candidate genes induced by AA or ATII implicated in oncogenesis: 3) to perform a similar analysis for p-AML and 4) to compare these two AML entities in our series and in the literature.

## Materials and Methods

### Patients

The patients were studied according to protocols approved by the Institut Gustave Roussy (Villejuif, France) Ethics Committee. Between 1995 and 2007, 30 t-AML and 36 p-AML with various karyotype results were collected at disease onset. (table 1 and 2).

**Table 1:** Clinical, biological features and karyotype of t-AML patients.

Pts^1^	Age^2^/	Primary ^3^ -	PC Tt^4^	FAB^5^	Karyotype at diagnosis of t-AML	FISH^6^	p-AML	AML	Allog.^9^	Survival>
	Sex	Cancer^3^ -					Type^7^	Tt^8^		12 month
**t-1**	71/F	Ovarian	Tax/SP/PSC833/Iressa	RAEB	57,XXX,+1,+4,del(5)(q15;q33)×2,−7,+8,+9,	MLL WT	A	no	no	no
		C.			+13,+14,−17,+18,+19,−20,+22,+mar [4]					
**t-2**	67/F	VLSL	Fluda/Chlorambucil	AML-M5	44, XX, 5q-, −7, −12, −14, −18,+ der(12?)	MLL WT	A	no	no	dead at
					+mar [20]					diagnosis
**t-3**	44/F	Breast C	Alk/AT II	AML-M0	46,XX,der(1)t(1;17)(p36;?),der(7)	MLL ND	U	yes	yes	yes
		Lipo-			t(7;?)(p15;?),der(10)(7;10;17)(?;p11;					
		sarcoma			q?21),der(17)(q11?),ins(17?) [11]					
**t-4**	45/F	Astrocy-,	RT/BCNU/Alk/	AML-M0	45,XX,dup(2)(q22;q34),-3p21,	MLL WT	U	yes	no	no
		toma	AT II, Autograft		t(5;13)(p15;q14),t(6;8)(p21;q24),+i(7q)					
		+ skin			,der(7)t(7;15)(q10;q10),der(12)					
		T NHL			t(12;17)(p13;q11),−15,−17 [18]					
**t-5**	72/M	Esophag.	5FU/SP/RT/curie	RAEB	45,XY,add(1)(q41),−5,−7, +mar [11]/	MLL ND	A	no	no	no
		C			45,XY,add(1)q41,−5,−7,					
					t(12;20?)(p12;q12),+mar [11]					
**t-6**	44/M	Follicular	ATII/Alk/Oncovin	AML	46,del(X)(q23),Y,der?(6),t(2;6)(q12;	MLL ND	A	no	no	no
		NHL	Alk/AT II/TBI/		q21),−7,del(18?)(q12),add(20)(q12)					
			Alk Fluda		but uncertain conclusion at diagnosis					
**t-7**	73/M	Multiple	Alk/steroids/INF/	AML +	46–**47**,XY,−7,−8,+r+dm [20]	MLL ND	A	no	no	no
		myeloma	AT II/BCNU	Myeloma						
**t-8**	72/M	Follicular	Alk/ATII/Fluda/	AML-M5	47,XY,del(7)(q21q32),+13 [11]	MLL ND	A	yes	no	no
		NHL	Chlorambucil,							
			Rituximab/							
			Spleen RT							
**t-9**	69/M	Head and	RT	AML-M5	45,XY, −7 [12]	MLL WT	A	no	no	no
		Neck C.								
**t-10**	57/M	Bladder	RT/SP	MDS	46,XY, [14]/46–47,XY,del(7q-) [5]	MLL ND	A	no	no	no
		Cancer				WCP 7				
**t-11**	62/M	Follicular	Alk/ATII/INF/	AML-M5	NA#	MLL ND	A	yes	no	no
		NHL	MethylGAG							
			Rituximab/skin RT							
**t-12**	63/M	NSCLC	AT II/SP/RT	AML	46 XY,t(8;21)(q22;q22) [1]/46 XY,	MLL ND	U	no	no	no
					t(8;21)(q22;q22),del(7)(q11q34) [13]					
**t-13**	38/F	Breast	Alk/RT/	AML	46,XX,add(1)(q 3?) [25]	MLL ND	U	yes	no	no
		Cancer	ATII/RT/AM							
**t-14**	25/F	Endometre	Alk/AT II/SP,	AML-M4	47,XX, +8 [14]	MLL WT	U	yes	yes	yes
		cancer	Actinomycine							
**t-15**	52/F	Breast	Alk/AT II/RT	biphenotypic	48,XX,+8,t(9;22)(q34;q11),+17 [12]	MLL ND	U	yes	no	no
		Cancer		AML-M4		WCP 8 +				
						WCP 17				
						BCR-ABL fus				
							T	yes	no	yes
							T	yes	no	yes
							T	yes	no	yes
**t-19**	55/F	Breast	Alk/ATII/RT/HT/	AML-M5	46,XX,t(9;11)(p21;q23) [21]	MLL ND	T	yes	no	no
		Cancer	Tax							
**t-20**	61/F	Breast C.	Alk/AT II/RT/HT	AML-M5	46,XX,t(9;11)(p22;q23) [14]	MLL sep	T	yes	yes	no
**t-21**	60/F	NHL	Alk/AT II, Autograft	AML-M5	46,XX,t(9;11)(p22;q23) [12]	MLL sep	T	yes	no	no
**t-22**	19/M	Osteo-	Alk/AT II/SP	AML-M5	46,XY,t(9;11)(p21;q23) [8]/	MLL sep	T	yes	no	yes
		sarcoma			46,XY,t(9;11)(p21;q23),+8 [10]					
**t-23**	22/F	HL	Alk/ATII/Bleo/	AML-M5	46,XX,t(11;19)(q23;q13) [12]	MLL sep	T	yes	no	dead at
			Oncov							diagnosis
**t-24**	44/F	Ovarian	Tax/SP/AT II/	biphenotypic AML	46,XX, [2]	MLL sep	T	yes	no	no
		cancer	Iressa							
**t-25**	64/F	Ovarian	Tax/SP/Iressa	MDS	46,XX [17]	MLL WT	U	no	no	no
		cancer								
**t-26**	51/F	Anal +	RT, Alk/AT II/RT	AML	46,XX [5]	MLL WT	U	yes	no	no
		Breast C								
**t-27**	47/F	Breast C.	Alk/AT II/RT/HT	AML-M2	46,XX [20]	MLL WT	U	yes	no	U
**t-28**	54/F	Breast C	Alk/AT II/RT	AML-M1	46,XX [16]	MLL WT	U	yes	yes	yes
**t-29**	71/M	Prostatic	RT	AML	46,XY [20]	MLL WT	U	no	no	no
		cancer.								
**t-30**	67/M	Head and	AT II/SP/Bleo/RT	AML-M5	46,XY [21]	MLL WT	U	yes	no	no
		Neck C.								

1 = patients. 2 = age at diagnosis of AML. 3 = Primary cancer; NHL: Follicular non hodgkin lymphoma, NSCLC: Non small cell lung cancer, VLSL: Villositary lymphocyte splenic lymphoma. 4 = Primary cancer treatment; Alk: alkylating agent, RT: radiotherapy, AT II: anti-topoisomerase II, AM: anti-metabolite, brachy: brachytherapy, Clo: Chlorambucyl, Tax: taxane, Fluda: fludarabine, SP: platinium, Bleo: bleomycin, HT: hormonotherapy, INF: interferon, Rit: Rituximab, RT: Radiotherapy, TBI: Total Body Irradiation, Oncov: Oncovin, A: alkylating-agent-inducing type, T: anti-topoisomerase-inducing type, U: undetermined. 5 = FAB classification: biph = biphenotypic. 6 = FISH column: MLL WT = MLL Wild Type, MLL R = MLL rearranged, ND = Not Done. 7 = Type of t-AML; A: alkylant induced, T anti-topoisomerase-induced. 8 = AML treatment. 9 = Allograft as treatment.

# This patient had secondary MDS for several years before the leukemia, with transitory partial monosomy 7, that had disappeared a year later, two years later the t-AML appeared.

**Table 2:** Clinical, biological features and karyotype of the p-AML patients.

Patients	Age^1^/	FAB	Karyotype	FISH	Allograft	Survival
	Sex					duration >
						12 months
**p-1**	68/F	M1	45,XX,−7	ND	No info	No info
**p-2**	34/M	M6	45,XY,−7	ND	no	no info
**p-3**	66/M	M1	48,XY,+8,+13	ND	No	Yes
**p-4**	54/F	M5	47,XX,+i(21q)	ND	Yes	Yes
**p-5**	32/M	M5	47,XY,t(2;14)(q21;q32),+4	wcp2, wcp14	No	No
**p-6**	44/F	M5	46,XX,der(5)t(1;5)(q32;q35),inv(9)(p12q13)[20].	MLL WT, wcp1,	No	No info
			ish(5p15,5q31)×2,add(5)(q35?)(wcp1+)	wcp5,wcp17		
**p-7**	68/M	M5	46,XY,del(7)(q21q31),der(9)(WPC9+),?der(11)(q23)/46,XY[2]	MLL WT	No	No
**p-8**	25/F	M3	46,XX,t(15;17)(q22;q21)	PML-RARA fus		
**p-9**	46/F	M3	46,XX,t(15;17)(q22;q21)	PML RARA fus	No	No info
**p-10**	46/F	M3	46,XX,t(15;17)(q22;q21)	PML RARA fus	No	Yes
**p-11**	27/M	M3	46,XY,i(3)(p10),i(3q),t(15;17)(q22;q21)	PML RARA fus	No	Yes
**p-12**	47/M	M3	46,XY,t(15;17)(q22;q21)	PML RARA fus	No	No
**p-13**	73/M	M3	46,XY,t(15;17)(q22;q21)	PML RARA fus	No	Yes
**p-14**	35/M	M3	46,XY,t(15;17)(q22;q21)	PML RARA fus	No	Yes
**p-15**	57/M	M3	46,XY,t(15;17)(q22;q21)	PML RARA fus	No	Yes
**p-16**	54/M	M4	46,XY,t(16;16)(p13;q22)	CBFB sep	Yes	No
**p-17**	26/M	M4Eo	46,XY,t(16;16)(p13;q22)	ND	Yes	Yes
**p-18**	19/M	M2	45,XY,t(8;21)(q22;q22),del(9)(q31q34)	ND	No	Yes
**p-19**	37/M	M1	45,XY,t(9;11)(p22;q23),del(7)(p12;p21)	MLL sep	No	Yes
**p-20**	25/M	M1	46,XY,t(9;11)(p22;q23)	MLL sep	Yes	Yes
**p-21**	80/M	M1	46,XY[3]/50–53,XY,−1,−3,−4,−15?,−21,+7 to 10 mar	ND	No	No
**p-22**	42/M	M1	46,XY,t(3;5)(q25;q34),del(4)(q26),der(12)t(4;12)	WCP(3; 4)	Yes	Yes
			(q28?;p12),der(18)t(12;18)(p12;q21),mar?	WCP(5; 12)		
**p-23**	30/M	M5	46,XY,t(11;12)(q13;p13)[2]46,XY,id,der(1)t(1;1)	ND	No	No
			(p16;q12)[1]/46,XY,id,der(7)t(1;7)(q12;q36)[1]/			
			46,XY,id,der(9)t(1;9)(q12;p24)[2]46,XY,id,der(14)			
			t(1;14)(q12;p10)[2]46,XY,id,der(20)t(1;20)(q12;p12)			
			[1]46,XY,id,der(20)t(1;20)(q12;q13)[2]46,XY,id,			
			der(21)t(1;21)(q12;q10)[3]			
**p-24**	71/M	M2	43–46,XY,−2,−5,−7,−16,+mar1,+mar2,+mar3, +mar4,	ND	No	No
			variations			
**p-25**	63/F	M2	46,XX	ND	No	Yes
**p-26**	52/F	M2	46,XX	MLL WT	Yes	Yes
**p-27**	23/F	M2	46,XX	MLL WT	No	Yes
**p-28**	23/F	M5	46,XX	MLL WT	Yes	Yes
**p-29**	45/F	M5	46,XX	ND	No	No
**p-30**	73/F	M5	46,XX	ND	No	No
**p-31**	31/M	M0	46,XY	MLL WT	No	No info
**p-32**	75/M	M1	46,XY	MLL WT	No	No
**p-33**	41/M	M1	46,XY	MLL WT	Yes	No info
**p-34**	48/M	M2	46,XY	MLL WT	Yes	Yes
**p-35**	38/M	M2	46,XY	MLL WT	No	No
**p-36**	63/M	M4	46,XY	ND	No	No

1 = age at diagnosis of AML. FAB column: FAB classification. FISH column: MLL WT = MLL Wild Type; MLL sep = MLL rearranged (separated); PML-RARA fus = fusion of PML and RARA gene; WCP = whole chromosome paint; ND = Not Done.

### Cytogenetic analysis

Cytogenetic analyses were performed on metaphase spreads obtained from tumor, bone marrow or blood. In each case, 20 RHG-banded metaphases were analyzed when possible.

### Fluorescence in situ hybridization (FISH) studies

A set of commercial probes was used to search for abnormalities such as WCP1, WCP2, WCP5, WCP7, WCP8, t(8;21) RUNX1-ETO fusion probes (fp), t(9;22)BCR-ABL (fp), MLL dissociation probes, WCP14, t(15;17) PML-RARA (fp), WCP17 (tables 1 and 2). They were used according to the manufacturer's protocol (Vysis® or Kreatech®).

### Cell culture

Most samples were diagnosis bone marrow cells except for patients t-4, t-9, t-11, t-17, t-19, t-26 and p-33 derived from whole blood. After thawing, all t-AML samples were cultured for 24h (20% fetal calf serum in RPMI 1640 with antibiotics) without selecting CD34+ cells. The mononuclear cells were collected and washed before DNA extraction. Bone marrow was cultured in the same flask with the same media, to allow adherent cell proliferation in order to obtain constitutional fibroblasts from the patient. However, no adherent cells were obtained from this procedure. Pooled commercial DNA (Promega®) was thus used for all t-AML and p-AML work in order to maintain a homogenous process for the 64 AML.

### Oligonucleotide aCGH

Tumor genomic DNA was isolated according to Qiagen protocols with modifications [5]. Samples containing more than 60% of blasts were chosen in order to analyze a majority of pathological cells. High-molecular-weight genomic DNA was extracted from the cell lines with a DNeasy extraction kit (Qiagen).

Patient leukemia samples were analyzed using 244 K microarrays (Agilent Technologies, Santa Clara, CA, USA). Only t-AML were processed as dye-swap pairs. In all experiments, sex-matched DNA from a pooled human female or male individual (Promega, Madison, WI) was used as the reference. Oligonucleotide aCGH processing was performed as detailed in the manufacturer's protocol (version 4.0; http://www.agilent.com). Data were extracted from scanned images using Feature extraction® software (version A.8.5.3, Agilent). Raw data text files from the latter were then imported for analysis into CGH Analytics® 3.4.40. Aberrations were detected with the ADM2 algorithm and the filtering options of a minimum of 5 probes and abs(log2Ratio) >0.3. Aberration segments were individually reviewed using build 35, hg18 from the UCSC Genome browser [6]. Anomalies that were localized to regions with high-copy repetitive or GC-rich DNA sequences including telomeric regions were excluded. We defined gains and losses for the oligonucleotide dataset as a linear ratio ≥1.2 or ≤0.8 respectively. High and low-level amplification events were defined as a linear ratio ≥4 or between <2 and <4 respectively. The data are described in accordance with MIAME guidelines and have been deposited in ArrayExpress under accession number E-TABM-1014.

## Results

### Copy Number Variations (CNV)

As no paired DNA had been obtained, Copy Number Variations (CNV) were distinguished from Copy Number Abnormalities (CNA) based on various criteria: i) sequence size below 2 Mb; ii) the presence of repetitive identical breakpoints between patients; iii) the genes involved such as olfactory receptor genes, the NF1P1 locus or GSTT1 (chromosome 22) and iv) consultation of the Database of Genomic Variants [7]. Many Mendelian CNVs, that are present throughout the entire genome [8], were seen in the two AML samples (table 3). Together with classic genes, four microRNA embedded in CNV regions were found: mir-570 in 3q29 within MUC20, mir-1268 in 15q11 within NF1P1, BCL8 and olfactory receptors, mir-1233 in 15q14 within GOLGCABB, and mir-1826 in 16p11.2 within TP53TG3 HERC2P4 (table 3).

**Table 3:** Germinal and immunoglobulin genes related CNVs.

Location	Some CNV genes	miRNA
Germinal CNVs		
−1p36.13 <16.71–17.14>	CROCC MSTP9 ESPNP	
−1q21.3 <150.82–150.84>	NLCE3C	
−1q23.3 <159.75–159.90>	FCGR2A FCGR2B FCGR3A HSPA6	
−1q44 <246.79–246.86>	OR2T34 OR2T10 OR2T11	
+/−3q29 <196.90–196.96>	MUC20	mir-570
+/−4q13.2 <69.05–69.16>	UGT2B17	
+5p15.33 <0.76–0.87>	ZDHHC11	
−6p25.3 <0.20–0.32>	DUSP22	
+/−6p21.2 <32.56–32.72	HLA-DQA1 HLA-DRB1 HLA-DRB5 HLA-DRB6	
+/−8p11.3 <39.35–39.50>	ADAM3A	
−10q11.22 <46.37–47.73>	ANXA8 PPYR1 GPRIN2 SYT15 ANTXRL	
+11q11 <55.12–55.20>	OR4P4 OR4C11 OR4S2 OR4C6	
+12p12 <19.36–19.46>	PLEKHA5	
+/−14q11.2 <18.62–19.49>	OR4K5 OR4K1 OR4N2 OR4K2 OR4Q3 OR4M1	
+/−15q11.2 <18.65–20.08>	BCL8 NF1P1 OR4N4 OR4M2	mir-1268
+/−15q14 <32.51–32.62>	GOLGABB	mir-1233
+/−16p11.2 <31.86–33.53>	TP53TG3 SLC6A10P HERC2P4…	mir-1826
+17q21.31 <41.52–41.57>	KIAA1267	
−17q21.31 <41.55–42.04>	NSF LRRC37A ARL17P1 KIAA1267 LRRC37A2	
−19q13.2 <48.20–48.43>	PSG1 PSG3 PSG6 PSG7 PSG11	
+/−22q11.23 <22.65–22.72	GSTT1.	
**Immunoglobulin genes related CNVs**		
−2p11.2 <89.10–89.26>	IGKC IGKV1-5 IGKV3D-15 IGKV1D-13 IGKV3-20 IGKV2-24	
+/−7p14.1<38.22–38.41>	TRGC2 TRGV5 TRGV7 TRGV9 STARD3NL	
+/−14q11.2 <21.43–22.04>	TRA@ TRD@ TRDV1 TRAV20 TRAJ17 TRAC	
−14q32.33 <105.13–106.00>	IGH@ IGHA1 IGHA2 IGHG1 IGHG2 IGHG4 IGHV4-31 C14orf81	

The CNVs are either lost or gained as indicated by a “−” or a “+”. Locations on chromosomes are described according to the ISCN 2009 with slight modifications: sequence numbers are included between <> and expressed in Mb, with a resolution of 10kb. Column 2: coding genes included in the CNVs: Column 3; miRNA genes included in the CNVs.

Deletions inside immunoglobulin gene (IG) clusters, consecutive to VDJ rearrangement were observed mainly in t-AML. These illegitimate recombinations were considered as acquired CNVs [5], characteristic of the malignant clone. In t-AML, four losses were located in 2p11 in IGLK and 9 in 14q32.3 in IGH. Two t-AML were bi-phenotypic acute leukemia (cases 2 and 7). In three cases (t-15, t-17 and t-25), both genes were rearranged (table 4). No relationships were observed with MLL or other translocations. In p-AML (table 5), only 2 rearrangements were observed: on IGLK (case p-21) and on IGH (case p-24). No rearrangement of IGLL on 22q11 was observed. The difference in the proportion of IG rearrangements in t-AML compared to p-AML was statistically significant with a p<0.002.

**Table 4:** Gains and losses CNA in t-AML and revised karyotype after aCGH.

Patients	Gains	Losses	Revised karyotypes
**t-2**	+14q11.2q12<24,09–26.24>[1.23]	−1q44<241.94–247.19>[0.61]	44,XX,**−1q44**,−5q14.3q35**,**−7,**−7q33q34,−12p11.2p13.2,**
	+14q12q13.3<29.52–35.74>[1.26]	−5q14.3q35<85.18–167.84>[0.58]	**−12p11.2q12,−12q13.1,−12q13.3q22,−12q23.3,**
	+14q21.1q21.2<41.52–44.69>[1.23]	−7pterp22.3<0.14–2.67>[0.65]	**−14q12,+14q12,−14q12,+14q12q13.3,**
	+14q21.3q22.1<47.25–52.51>[1.54]	−7p21.3<8.21–11.79>[0.64]	**−14q21.2,+14q21.1q21.2,−14q21.2,+14q21.3q22.1,**
		−7p15.2q14.3<27.40–33.28>[0.67]	**−18q11.1qter,−18q11.2q12.1,**
		−7p14.1q33<39.89–135.74>[0.63]	**−18q22.3q23**
		***−7q33q34<135.74–137.48>[0.16]***	
		−7q34qter<137.48–158.76>[0.60]	
		−12p13p11.2<11.27–25.37>[0.61]	
		−12p11.2q12<32.77–37.09>[0.65]	
		−12q13.1<47.09–49.44>[0.66]	
		−12q13.3q22<55.30–92.75>[0.63]	
		−12q23.3<105.58–107.51>[0.68]	
		−14q11.2<23.73–24.07>[0.70]	
		−14q12<26.25–27.10>[0.65]	
		−14q21.2<35.77–37.62>[0.66]	
		−14q21.2<44.69–45.26>[0.66]	
		−18q11.1q11.2<16.79–21.74>[0.59]	
		***−18q11.2q12.1<21.74–24.41>[0.22]***	
		−18q12.1q22.3<24.41–71.05>[0.59]	
		***−18q22.3q23<71.06–71.76>[0.23]***	
		−18q23<71.76–76.11>[0.59]	
**t-24**	+17q11.1qter<22.80–78.65>[1.39]	−2p11<89.65–91.05>[0.62]*	46,XX**,**t(11;?)(q23;?)**,i(17)(q10),−19p13.3,+19p13.3**
	+19p13.3<4.96–6.82>[1.44]	−17pterp11.2<0.28–21.22>[0.63]	
		−19p13.3<0.21–4.96>[0.60]	
**t-8**	+13q11qter<18.36–114.10>[1.46]	−7q31.1q36.1<107.67–150.28>[0.55]	47,XY,**−Xp11.4,−7q31.1q36.1,**+13,**−21q22.1**
		***−21q22.1<35.01–35.60>[0.11]***	
		***−Xp11.4<39.25–39.85>[0.16]***	
**t-13**	+2p16.1p25.3<0.02–54.88>[1.46]	−1q42.3.q44<233.13–247.19>[0.53]	46,XX**,der(1)t(1;2)(q42.3;p16.1),−10q11.2**
		−10q11.2<45.53–47.97>[0.78]	
**t-30**		−2p23.3<25.31–25.60>[0.50]	46,XY,**−2p23.3**
		−14q32.3<105.96–106.00>[0.65]*	
**t-15**	+8pterqter<0.16–146.26>[1.31]	−2p11<88.98–91.05>[0.46]*	46,XX,+8,t(9;22)(q34;q11),+17
	+17pterqter<0.02–78.65>[1.31]	−4q31.2<146.62–146.67>[0.68]	
		−14q32.3<105.40–106.15>[0.34]*	
**t-4**	+2q14.3q32.3<128.11–193.87>[1.34]	−1p36.11<26.73–27.04>[0.64]	**46,**XX,**dup(2)(q14.3q32.3),**t(5;13)(p15;q14)
	+7q11.2q36.3<61.46–158.81>[1.65]	−1q43<237.92–238.50>[0.62]	**−5q31.3q32,**+i(7)(q10)**,der(7)t(7;15)(q10;q21.3),**
	+12p12.1 p11.2<24.32–30.60>[1.37]	−5q31.1q33.1<142.91–148.19>[0.63]	**der(12)t(12;17)(p12.1;q11),+12p11.2p12.1,**
		−7p11.1p22.3<0.14–57.89>[0.65]	**−15pterq21.2, −17p11.2p13.3,−17q23.2**
		−12pterp12.1<0.05–21.96>[0.63]	
		−14q32.3<105.13–105.84>[0.68]*	
		−15q11.2q21.3<20.24–55.80>[0.65]	
		−17p13.3p11.2<0.02–22.07>[0.65]	
		−17q22<51.84–54.75>[0.64]	
**t-16**	+7p15.2<27.15–27.18>[1.33]		46,XX,**+7p15.2**,**+8q24.2q24.3,**inv(16)(p13;q22)
	+8q24.2q24.3<139.48–140.18>[1.46]		
**t-23**	+4q35.1q35.2<187.17–187.37>[1.49]	−14q32.3<106.21–106.25>[0.66]*	46,XX,**+4q35.1q35.2,**t(11;19)(q23;q13)
**t-17**	+7p15.2<27.15–27.18>[1.26]	−2p11<89.10–89.89>[0.70]*	**−3p12.3p14.1,+7p15,**t(15;17)(q22;q21)^+^
	+6q27<168.12–168.84>[1.20]	−3p12.3p14.1<69.07–79.19>[0.54]	.ish(PML;RARA)
		−14q32.3<105.85–105.89>[0.59]*	
**t-29**		−14q32.3<105.94–106.00>[0.66]*	46,XY,−**21q22.1**
		−21q22.1<34.98–35.29>[0.59]	
**t-3**	+8q24.3<143.73–143.90>[1.50]	−14q32.3<104.96–105.22>[0.76]*	46,XX,**t(1;17)(p36;?),t(7;?)(p15;?),+8q24.3,**
			**t(7;10;17)(?;p11;q21;?)**
**t-26**	+2q11.2<98.43–98.63>[1.42]		46,XX,**+2q11.2**
**t-12**	+21q22.1qter<35.07–46.91>[1.19]	−7q22.1q36.3<101.85–158.79>[0.80]	46,XY,−7q21.2q36.3**,**t(8;21)(q22;q22),**+21q22.1qter**
**t-9**	+4p16.3<1.20–1.30>[1.38]	−7pterqter<0.14–158.76>[0.56]	45,XY,**+4p16.3,**−7
	+6q27<168.08–168.32>[1.50]		
**t-11**		−21q22.12<35.11–35.15>[0.68]	46,XY,**−21q22.12**
**t-14**	+7p15.2<27.15–27.18>[1.38]	−14q32.3<105.96–106.00>[0.60]*	47,XX,**+7p15**,+8,−17q11.2
	+8pterq24.3<0.16–142.23>[1.35]	−17q11.2<25.96–27.38>[0.72]	
	+8q24.3<142.23–145.81>[1.52]		
	+8q24.3<145.81–146.26>[1.35]		
**t-1**		−5q15qter<94.36–177.87[0.79]	57,XXX,+1,+4,del(5)(q15q33)×2,−7,+8,+9,+der(11),
			+13, +14,−17,+18,+19, −20,+22,+mar
**t-5**	+5p15.2p14.3<10.78–21.71>[1.27]	−3p14.1p12.3<69.67–80.18>[0.54]	**46,**XY,**−3p12.2p14.1,−5p15.2pter,**+5 p14.3p15.2,
	+5p13.3q11.2<31.24–53.59>[1.31]	−5pterp15.2<0.09–10.76>[0.57]	**+5p12q12.3,−5q12.3qter,−7p11.1p22.3,**
	+21q22.1<36.40–36.53>[1.60]	−5q12.3qter<65.23–180.64>[0.57]	**−7q21.3q32.1,−12p13.1p13.2,+21q22.1**
		−7pterp11.1<0.14–57.66>[0.57]	
		−7q21.3q36.3<97.13–158.76>[0.57]	
		−12p13.2p13.1<11.82–13.33>[0.62]	
**t-10**	+1q44<246.44–247.06>[1.32]	−7p22.2<3.02–3.12>[0.48]	46,XY,**+1q44,−7p22.2,−7q11.2,−7q21.3q22.1**
		−7q11.2<64.40–75.85>[0.77]	
		−7q21.3q22.1<97.69–101.88>[0.77]	
**t-7**	+9q34.3<135.98–139.80>[1.30]	−7pterqter<0.14–158.81>[0.79]	**45,**XY,−7,**+9q34.3,**+**12p13.1**,+**12p11.1,**−**12q11q12**,-
	+12p13.1<14.25–14.61>[1.36]	−12q12<36.73–42.14>[0.65]	**−12q13.1,+12q13.12q14.3,−17p11.2p13.3,**
	+12p11.1<31.55–34.64>[1.34]	−12q13.1<45.33–47.69>[0.68]	**−21q21.1q21.2,+21q21.2q21.3,−21q22.11q22.12,**
	+12q13.1q14.3<47.69–51.83>[1.42]	−17pterp11.3<0.02–21.23>[0.78]	**+21q22.12q23,−21q22.3**
	+12q14.1q14.3<58.80–64.31>[1.33]	−21q21.1q21.2<19.84–23.05>[0.65]	
	+21q21.2q21.3<23.17–30.35>[1.42]	−21q22.1<34.38–36.12>[0.66]	
	+21q22.1q22.3<36.20–43.01>[1.58]	−21q22.3<43.02–43.08>[0.65]	
**t-6**		−7pterqter<0.14–158.76>[0.70]	45,XY,t(2;6)(q12;q21),−7
**t-22**	+2p25.2<2.54–3.57>[1.37]		46,XY**,+2p25.3,**+8,t(9;11)(p21;q23)
	+8pterqter<0.16–146.26>[1.40]		
**t-19**	+7p15.2<27.15–27.18>[1.40]	No CNA	46,XX,+7p15.2,t(9;11)(p22;q23)
**t-21**	No CNA	No CNA	46,XX,t(9;11)(p22;q23)
**t-20**	No CNA	No CNA	46,XX,t(11;19(p22;q23)
**t-18**	No CNA	−14q32.3<105.94–106.00>[0.64]*	46,XX,t(15;17)(q22;q21)
**t-25**	No CNA	−2p11<89.10–91.05>[0.69]*.	46,XX
		−14q32.3<105.96–106.00>[0.61]*	
**t-27**	No CNA	No CNA	46,XX
**t-28**	No CNA	No CNA	46,XX

Column 2 and 3: The CNA are either lost or gained as indicated by a “−” or a “+”; locations on chromosomes are described according to the ISCN 2009 with slight modifications: sequence numbers are included between <> and expressed in Mb, with a resolution of 10kb; linear ratios are written between brackets after an “×”; CNAs with a linear ratio >2 (low level of amplification) or losses <0.25 are labeled in **bold** and ***italics***; “*” indicates CNA that are probably part of a rearrangement of the immunoglobulin genes. They have not been included in the synthetic karyotypes because they could be considered as an acquired CNV which is characteristic of monoclonal proliferation.

Column 4: In **bold** are new data or those modified by aCGH in the synthetic karyotypes; CNAs that were contiguous but whose ratios were not too different were fused to express overall chromosome abnormality for readability.

**Table 5:** Gains and losses CNA in p-AML and revised karyotype.

Patients	Gains	Losses	Revised karyotypes
**p-24**	+1p36.3<1.52–7.56>[1.26]	−2pterp22.3<0.02–32.60>[0.66]	**46,XY,+1p34.2p36.3,**−2pterp22.3**,**−2p14p13.3,
	+1q21q22<153.19–154.64>[1.67]	−2p14p13.3<68.01–71.72>[0.66]	−2q35qter,**+3p21.3, −3p21.3p22.2,+4p16.3,**
	**+3p21.3<50.26–50.29>[2.54]**	−2q35qter<219.02–242.39>[0.66]	−5**q14.2q33.3,−7p15.2p15.3,**−**7q11.2,**−**7q21.1q22.2,**
	**+3p21.3<52.29–52.31>[4.00]**	−3p22p21.3<38.35–49.66>[0.66]	**−7q31.3q36.3,+15q11.2,+15q22.2q26.2,**
	+4p16.3<0.99–1.65>[1.61]	−5q14.2q33.3<82.18–158.75>[0.61]	**+15q23,−15q26.2q26.3,−16p13.3q23.1,**
	**+15q22.2q23<56.84.18–67.10>[2.05]**	−7p15.2p15.3<19.64–26.31>[0.63]	**+16q23.3q24.3,−17p11.2q12,−17q21.31q21.3,**
	**+15q23<67.44–68.37>[3.64]**	−7q11.22q11.23<69.84–72.44>[0.65]	**+19p13.3p13.1, +20p12.3p13, +21q22.1q22.3,+22q13.1**
	**+15q24.1<70.42–72.67>[2.89]**	−7q21.12q22.2<87.65–105.96>[0.71]	
	+15q24.1q26.2<72.70–94.54>[1.30]	−7q31.32q36.3<123.35–158.81>[0.64]	
	+16q23.3q24.3<82.61–88.67>[1.8]	−14q32.3<105.60−105-70>[0.74]*	
	+19p13.3p13.1<0.64–19.72>[1.41]	−15q26.2q26.3<94.55–100.21>[0.75]	
	+20p12.3p13<0.27–9.14>[1.26]	−16p13.3q23.1<15.38–78.80>[0.74]	
	**+21q22.1<33.30–37.23>[1.79]**	−17p11.2q12<0.18–29.54>[0.65]	
	**+21q22.1<37.24–38.54>[2.5]**	−17q21.3q21.33<38.29–46.94>[0.66]	
	**+21q22.1<38.54–39.38>[3.00]**		
	**+21q22.2q22.3<39.40–43.22>[2.18]**		
	+21q22.3<43.23–46.89>[1.70]		
	+22q13.1<35.86 = 37.20>[1.33]		
**p-21**	+3p14.3p14.2<57.97–62.19>[1.48]	−1q21.1q44<147.21–247.19>[0.62]	50–53,XY,−1**q21.1q44,−2p24.3,**
	+3p14.2p14.1<63.60–64.29>[1.31]	−2p11.2<89.38–91.05>[0.61]*	−3**p12.1p14.1,+3p14.1,−3p14.1,+3p14.1p14.2,**
	**+3p14.1<64.32–65.50>[4.61]**	−2pterp24.3<0.02–16.22>[0.61]	**+3p14.2p14.3,−3p14.3p24.3,+3p11.2qter,**
	**+3p14.1<66.40–67.67>[4.71]**	−3 p24.3p14.3<0.03–57.40>[0.63]	**−4q22.1q27,−9q22.3q31.1,**
	+3p11.2qter<89.28–199.32>[1.35]	−3p14.2<62.20–63.58>[0.60]	**+21q11.2q21.1,**
	**+21q11.2q21.1<14.29–17.97>[5.46]**	−3p14.1<65.51–66.11>[0.60]	**+21q22.1qter**
	**+21q22.1q22.3<38.27–43.54>[5.45]**	−3p14.1p12.1<67.68–86.30>[0.67]	
	**+21q22.3<43.59–45.12>[2.11]**	−4q22.1q27<88.94–123.72>[0.61]	
	+21q22.3qter<45.12–46.91>[1.7]	−9q22.3q31.1<93.92–103.86>[0.63]	
**p-22**		−4q28.2q28.3<130,24–134,36>[0,55]	46,XY,t(3;5)(q25;q34),**del(4)(q28.2q28.3),**
		−12p12.1p13.2<11,40–21,20>[0,55]	der(12)t(4;12;18)(q28?;p12;q21)**,**
		−18q21.2<48,99–51,05>[0,55]	**−12p12.1p13.2**,**−18q21.2,−18q22.3q23**
		−18q22.3q23<69,56–76,03>[0,55]	
**p-23**	+1q23.2qter<158.48–247.17>[1.24]	No CNA	46,XY, t(11;12)(q13;p13)/46,XY,id,der(1)t(1;1)(p16;q12)/
			46,XY,id,der(7)t(1;7)(q12;q36)/
			46,XY,id,der(9)t(1;9)(q12;p24)/46,XY,id,
			der(14)t(1;14)(q12;p10)/46,XY,id,der(20)t(1;20)
			(q12;p12)/46,XY,id,der(20)t(1;20)(q12;q13)/
			46,XY,id,der(21)t(1;21)(q12;q10),+1q23.2qter
**p-5**	+4pterqter<0.06–191.02>[1.43]	No CNA	47,XY,t(2;14)(q21;q32),+4
**p-32**		−3p21.3p21.3<44,20–49,02>[0,59]	46,XY,**−3p21.3**
**p-6**	+1q32.1qter<201.31–247.10>[1.39]		46,XX,der(5)t(1;5)(q32;q35),inv(9)(p12q13)
**p-1**		−7<0.14–158.76>[0.61]	45,XX,−7
**p-2**	**+Xp22.2<15.95–16.61>[2.04]**	−7<0.14–158.76>[0.55]	45,XY,**+Xp22.2,**−7,−22q11.2
		−22q11.2<21.36–21.41>[0.52]	
**p-7**	+11q24.2qter<124.85–134.43>[1.23]	−7q31.2qter<115.49–158.78>[0.77]	46,XY,−7**q31.2q36.3, +11q24.2q25**
**p-3**	+8pterqter<0.16–146.25>[1.27]	−2p11.2<88.93–89.14>[0.69]*	48,XY,+8,+13
	+13pterqter<18.31–114.12>[1.28]		
**p-18**	No CNA	−8q21.3q22.1<93.15–94.17>[0.63]	46,XY,t(8;21)(q22;q22),**del(8)(q21.3q22.1)**,**−9q12q31.1**
		−9q12q31.1<67.20–104.45>[0.62]	
**p-13**	+17q21.2<35.76–35.84>[1.49]	No CNA	46,XY,t(15;17)(q22;q21),**+17q21.2**
**p-4**	+21q11.2qter<14.31–46.91>[1.77]	No CNA	47,XX,+i(21q)
**p-8**	No CNA	No CNA	46,XX,t(15;17)(q22;q21)
**p-9**	No CNA	No CNA	46,XX,t(15;17)(q22 ;q21)
**p-10**	No CNA	No CNA	46,XY,t(15;17)(q22;q21)
**p-11**	No CNA	No CNA	46,XY,t(15;17)(q22;q21)
**p-12**	No CNA	No CNA	46,XY,t(15;17)(q22;q21)
**p-14**	No CNA	No CNA	46,XY,t(15;17)(q22;q21)
**p-15**	No CNA	No CNA	46,XY,t(15;17)(q22;q21)
**p-16**	No CNA	No CNA	46,XY,t(16;16)(p13;q22)
**p-17**	No CNA	No CNA	46,XY,t(16;16)(p13;q22)
**p-19**	No CNA	−14q32.3<105.02–105.04>[0.76]*	45,t(9;11)(p22;q23),del(7)(p12;p21)
**p-20**	No CNA	No CNA	46,XY, t(9;11)(p22;q23)
**p-25**	No CNA	−14q32.3<105.96–105.94>[0.61]*	46,XX
**p-26**	No CNA	No CNA	46,XX
**p-27**	No CNA	No CNA	46,XX
**p-28**	No CNA	No CNA	46,XX
**p-29**	No CNA	No CNA	46,XX
**p-30**	No CNA	No CNA	46,XX
**p-31**	No CNA	No CNA	46,XY
**p-33**	No CNA	No CNA	46,XY
**p-34**	No CNA	No CNA	46,XY
**p-35**	No CNA	No CNA	46,XY
**p-36**	No CNA	No CNA	46,XY

Column 2 and 3: The CNA were either lost or gained as indicated by a “−” or a “+”; the locations on the chromosomes are described according to the ISCN 2009 with slight modifications: sequence numbers are included between <> and expressed in Mb, with a resolution of 10kb; linear ratios are written between brackets after an “×”; CNAs with a linear ratio >2 (low level of amplification) or losses <0.25 are labeled in **bold** and ***italics***; “*” indicates CNA that are probably part of a rearrangement of the immunoglobulin genes. They are not included in the synthetic karyotypes as they could be considered as an acquired CNV characteristic of monoclonal proliferation.

Column 4: In **bold** are new data or those modified by aCGH in the synthetic karyotypes; CNAs that were contiguous but whose ratios were not too different were fused to express overall chromosome abnormality for readability.

### Karyotypes and CNA

**Figure 1:**
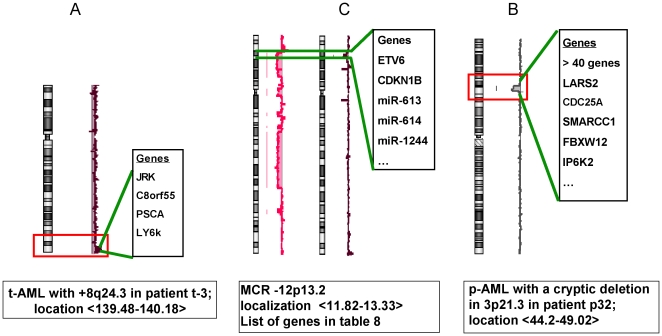
Details of critical rearrangements concerning MCRs. A = gain on 8q24.3 in a t-AML; B = loss of 12 p13 in t-AML (patients t-4 and t-5); C loss of 3p21.3 in a p-AML.

Karyotypes and aCGH were well correlated, with few exceptions. Ninety-six unbalanced chromosomal abnormalities previously undetected by mitotic karyotypes, were detected after high resolution aCGH (tables 1, 2, 4 and 5). Most of the additional abnormalities were too small to be detected by karyotypes. Some revealed masked rearrangements (Figure 1). In case t-1, the discrepancy between the karyotype and aCGH was probably due to a combination of hyperploidy, genetic heterogeneity and the presence of normal cells resulting in aCGH detection of only the del(5q).

In both groups of AML, 25 cytogenetically balanced translocations (table 1, 2 tables 4 and 5) did not show any cryptic rearrangement (gain or loss) at their breakpoint locations. In 5 cases (t-3, t-5, t-12, p-18, p-13), a CNA was found near the breakpoints. Patient t-3 was considered as having multiple balanced translocations that could not be further analyzed due to insufficient material. Patient t-5 had a poorly defined translocation implicating 12p and according to aCGH analysis, a small loss began in the middle of ETV6, suggesting a rearrangement of this gene. Patient t-12, had a standard t(8;21) and following “good practice” rules, no further investigation was initially performed. aCGH revealed a duplication of 21q22.1qter that was confirmed by FISH as another translocation or insertion on an unidentified chromosome. The unbalanced translocation breakpoint was 30 Kb centromeric to the 3′ end of RUNX1 suggesting a possible double event. Patient p-18, with a t(8;21) p-AML, had a 1Mb loss that included the first two exons of RUNXT1 on 8q21.3. Patient p13 had a t (15.17) accompanied by a small duplication of the telomeric part of RARA and of the TOP2A genes (table 5).

In some cases the confrontation between aCGH and the karyotype allowed us to better define the previously diagnosed rearrangements. In patient t-13, an add(1)(q3?) was found in the morphological karyotype. With aCGH, it was concluded that it resulted from an unbalanced translocation der(1)t(1;2)(q42.3;p16.1). The breakpoint was virtually cloned from the arrays: on 1q42.3<231.374>, it was sitting in the middle of PCNXL2, between exons 11 and 12. On 2p16.1<54.943>, it was lying in an EML6 gene in the vicinity of exon 12 of the gene. P-22, a primary AML with a t(3;5), had a loss of 10Mb of 12p13 secondary to a additional complex translocation encompassing ETV6 and CDKN1B (table 5).

### t-AML

All patients (excepted three who had received radiotherapy alone) had been treated with multi-agent chemotherapy (AA and ATII) combined or not with radiotherapy. Thus, the type of induction mechanism was most of the time, deduced from the observed chromosomal abnormalities (table 1): 9 cases were probably AA induced, 9 were ATII-induced and in 12, the mechanism could not be determined because CNA categorization was indistinguishable between t-AML induced by AA or by ATII. The medium time elapsed between cancer therapy and the diagnosis of t-AML was 5.1 years, 2.3 years and 4.7 years respectively.

Twenty-five patients (80%) with 104 CNA exhibited 41 gains and 63 losses (table 4). The mean number per total case number was 3.46. Six patients with a normal karyotype had at least one CNA. Six patients (20%) had no CNA, 3 had a normal karyotype and the 2 others had a balanced translocation (one a t(15;17) and one a MLL rearrangement). The mean CNA length was 4.1Mb for the t-AML patients with at least an unbalanced rearrangement.

The twelve losses in the immunoglobulin genes (IG) on 2p11 and 14q32.3 were considered as a special category of CNA. Among the 8 patients with IG rearrangements, 2 had bi-phenotypic leukemia and the others had various forms of AML.

### p-AML

Among 36 patients, 12 had a normal karyotype, 4 exhibited an anomaly on chromosomes 5 or 7. A t(15;17)(q22;q21), characterizing AML-M3, was present in 9 cases (table 2).

In p-AML, 64 CNAs were observed (table 5) with 30 losses and 34 gains while the mean number was 1.78. Twenty-two patients had no CNA, 11 had a recurrent balanced translocation while the 11 others had a normal karyotype. The mean number of CNA among p-AML patients with unbalanced rearrangements was 2.66. This value was due to 4 patients with very complex genomic rearrangements (>8 chromosomal abnormalities).

### Minimal Critical Regions (MCR)

A MCR was defined as such if 2 cases or more shared a common genomic location. Twelve MCRs were observed in t-AML and eight in p-AML (table 6).

**Table 6:** Minimal Critical Region in the two groups of patients.

MCR (localization by aCGH)	Patients	Size (Mb)	Possible genes
t-AML			
−1q44 <241.94–247.19>	t-2, t-13	5.25	>25 genes among them SMYD3
−3p14.1p12.3 <69.67–79.19>	t-5, t-17	9.52	MITF FOXP1 EIF4E3 GRP27 PROK2 RYBP
			SHQ1 PPP4R2 PDZRN3 CNTN3 FAM86D
			FRG2C ZNF717 ROBO2 ROBO1
−5q31.3q33.1 <142.91–148.19>	t-1, t-2, t-4, t-5	5.28	ADRB2 DPYSL3 HTR44 POU4F3 PPP2R2B
			SPINK1 JAKMIP2 TCERG1 SPINK5 LARS
			RBM27 KCTD16 HMHB1 FBXO38 YIPF5
			SPINK7SCGB3A2 GPR151 PRELID2
			SH3RF2 PLAC8L1 STK32A SPINK6 SPINK9
+6q27<168.12–168.32>[1.50]	t-9, t-17	0.20	KIF25 FRMD1 HGC 6.1.1
−7p22.2 <3.02–3.12>	t-5, t-6, t-7, t-9, **t-10**	0.10	GNA12 CARD11
+7p15.2 <27,15–27,18>	t-14, t-16, t-17, t-19	0,03	HOXA6 HOXA7 HOXA9 HOXA10 miR-196b
−7q21.3 <97.69–101.88>	t-2, t-5, t-6, t-7, t-9,	4.19	NPTX2 LMTK2 BRI3 BAIAP2L1 BHLHB8 CUX1
	t-10		miR-86 miR-93miR-106b miR-548o
−7q33q34<135.74–137.48>	**t-2**, t-5, t-6, t-7, t-8, t-9	1.74	CHMR2 PTN DGK1 CREB3L2 miR-490
+8q24.3 <143.73–143.9>	**t-3**, t-14	0.17	PSCA LY6D JRK C8orf55 LY6K
			SLURP1 LYNX1 LYPD2
−12p13.2p13.1<11.82–13.33>	t-2, t-4, **t-5**	1.51	ETV6 CDKN1B CREBL2 EMP1 GPR19 LRP6
			GPRC5A HEBP1DDX47 MANSC1 GPRC5D
			KIAA1467 BCL2L14 DUSP16 APOLD1 GSG1
			HTR7P miR-613 miR-614 miR1244
+17q21.3 <22.80–78.65>	t-15, **t-24**	55.85	Whole 17q
−21q22.1 <35.11–35.15>	t-7, t-8, **t-11**, t-29	0.04	RUNX1
			
**p-AML**			
+1q21q22<153.19–154.64>	p-23, p-24	1.4	>25 genes EFNA4 R1AP1 DPM3
			YY1AP1 DAP3 ROBLD3 CCT3 CLK2
+1q32.1q44<201.31–247.19>	p-6, p-23	45.69	Numerous genes
−3p21.3<44.20–49.02>	p-21, p-24, p-32	5.18	>40 genes SMARCC1 NCKIPSD
−7q31.2q36.3<115.49–158.78>	p-1 and p-2, p-7	43.29	Numerous genes
−9q22.3q31.1<93.92–103.96>	p-18, p-21	9.81	>60genes FANCC XPA
**Amp 21q21.1<14.29–17.97> [5.46]**	p-4, p-21	3.78	LIPI ABCC13 RBM11 HSPA13 SAMSN1
			NRIP1 USP25 C21orf34 CXADR BTG3
			miR-99a miR-125b-2hsa-let-7c
**Amp 21q22.2<38.27–43.54> [3 & 5.2]**	p-4, p-21, p-24	5,25	DSCR4 KCNJ15 ERG ETS2 BRWD1
			other genes
+21q22.3qter<43.54–46.91>	p-4, p-21, p-24	7.49	>50 genes

Column 1, the location of the MCR that follows the rules of tables 4 and 5; the figures in brackets and in bold are the ratios of the amplified regions. Column 2, patients; the figures in bold indicate the smaller CNA.

The size of MCRs was smaller in t-AML, with a minimum of 0.04 Mb compared to p-AML with a minimum of 0.95 Mb. The longest MCR in t-AML was 36.44 Mb, of the same order of magnitude as in p-AML where it was 45.69 Mb.

### Unique CNA

These single cases were of various sizes, from 0.04Mb to 26.89 Mb. Most of them were small and not known as polymorphisms. They are detailed in table 7.

**Table 7:** Single CNA of interest.

Patients	MCR unic cases	Size (Mb)	Possible genes
t-AML			
**t-2**	+14q22.1 <47.25–52.51>	5.26	>30 genes
**t-2**	−18q11.2q12.1<21.74–24.41>[0.23]	2.67	SS18 (Synovialosarcoma translocation) +7 other genes
**t-2**	−18q22.3q23<71.06–71.76>	0.69	TSHZ1 Colon cancer ag a zinc finger protein
**t-13**	−1q42.3.q44<233.13–247.19>	14.06	>100 genes
**t-13**	−10q11.2<45.53–47.97>	2.44	>20 genes
**t-5**	−12p13.2p13.1<11.82–13.33>	1.51	>20 genes ETV6 CDKN1B miR613 miR614 miR1244
**t-22**	+2p25.3 <2.54–3.57>	1.03	TSSC1 TTC15 ADI1 RNASEH1
**t-14**	−17q11.2 <25.96–27.38>	1.42	EVI2A EVI2B NF1 OMG SH3GLP2 SUZ12 CRLF3 C17orf79
			CENTA2 UTP6 C17orf42 ATAD5 RNF135 RAB11FIP4
			LRRC37B SUZ12P miR193a miR365-2
**t-15**	−4q31.2 <146.62–146.67>	0.05	SMAD1
**t-30**	−2p23.3<25.31–25.60>	0,10	DNMT3A miR1301
**p-AML**			
**p-24**	+3p21.3<50.26–50.29>[2.54]	0.03	GNAI2 SEMA3B
**p-24**	+3p21.3<52.29–52.31>[4.00]	0.03	NCLYTK miR135a-1
**p-21**	+3p14.1<64.32–67.67>[4.59]	1.94	LR1G1
**p-18**	−8q21.3q22.1<93.15–94.17>[0.63]	1.02	RUNXT1
**p-22**	−12p12.1p13.2<11.40–21.20>[0.55]	9.8	ETV6 CDKN1B among more than 20 genes
**p-24**	−17p11.2q12<0.18–29.54>[0.65]	29,36	multiple genes
**p-24**	+15q23<67.44–68.37>[3.64]	3.31	KIF23 RPLP1 TLE3 miR-629
**p-22**	−18q21.2<48.99–51.05>[0.55]	2.06	DCC and 4 other genes

Column 2: The CNA were either lost or gained as indicated by a “−” or a “+”; the locations on chromosomes are described following the ISCN 2009 with slight modifications: sequence numbers are included between <> and expressed in Mb, with a resolution of 10kb; linear ratios are written between brackets after an “×”; chromosomes with a linear ratio >2 (low level of amplification) or losses <0.25 are labeled in **bold** and ***italics***.

## Discussion

### The CNVs

As we were obliged to use a pool of normal DNA as a reference, Mendelian CNVs that are present throughout the entire genome [8], were revealed by the 244K aCGH (table 3). They were identified in the Toronto Database [7] in order to avoid including them in the analysis of somatic CNA. However, as a relationship between some CNVs and malignancy has already been suggested [9], several of them were retained in a separate table (table 3). For example a GSTT gene cluster was reported to be a susceptibility co-factor in cancer [10], [11], [12]. It is noteworthy that several miRNA, i.e. mir-570, mir-1268, mir-1233 and mir-1826 were found embedded in CNVs, with a possible multigene regulatory effect, as reported by Lin [13].

High resolution aCGH allowed us to study the whole set of IG gene rearrangements [5]. VDJ rearrangement in immunoglobulin genes (IG) has been described in AML [14], [15] and associated with MLL rearrangements [16]. The two series of patients (t-AML *vs* p-AML), were found to be significantly different, with a p value of <0.002. The fact that a quarter (8/30) of t-AML exhibited a VDJ rearrangement suggests that the transformed cells are often multipotent in these diseases.

### The CNAs

The acquired chromosomal abnormalities detected by morphological cytogenetics were quite similar in t-AML and p-AML. The CNA patterns were well correlated between the morphological cytogenetics and aCGH. Their frequency was increased after high resolution aCGH. Losses were more frequent than gains in both series. The number of CNAs was higher in t-AML (n = 104, mean number 3.5), than in p-AML (n = 69, mean = 1.9). The value found here is of the same order of magnitude than that reported elsewhere [17], [18], [19], [20]. Subcytogenetic CNA were present in half of the t-AML with normal karyotypes (table 4) while only 1 case (p-32) was observed in p-AML (tables 5 and 7). These findings are in accordance with the role of cytotoxic drugs in oncogenesis in t-AML [4], [21].

Twenty-five balanced translocations remained undetected (tables 4 and 5). No deletion or amplification at the breakpoint was observed in the five t-AML, nor in the two p-AML cases with rearranged MLL. The exceptions were two t(8;21) complex translocations resulting in detectable quantitative CNA at their breakpoints. In case t-12, a t-AML, a distal trisomy 21 began at the 5′ extreme part of RUNX1 (chr21:35.08). FISH studies showed a distal 21q unbalanced translocation on an unidentified chromosome in addition to the classic t(8;21) derivatives. In case p-18, a p-AML with a t(8;21), a 1Mb loss on 8q21.3 amputated the first 5′ exons of RUNX1T1, probably inactivating the ETO-AML1 chimeric gene on the der(21) and left the 3′ RUNX1T1 coding sequence intact in the AML1-ETO transcript on the der(8) [22]. This cryptic rearrangement allowed a “virtual cloning” of the t(8;21). The detailed molecular mechanisms of these rearrangements with an apparently identical t(8;21) seems to be different in these secondary and *de novo* leukemias.

### MCR

In the present series, twelve MCRs were isolated in t-AML and 8 in p-AML (table 6). The size of MCRs was smaller in t-AML, with a minimum of 0.04Mb compared to 1.4 Mb in p-AML. There were 7 MCR of <2Mb in p-AML compared to only 1 in p-AML. The longest MCR in t-AML was 55 Mb, of the same order of magnitude as in p-AML where it was 45.69 Mb. A quarter of MCR contained sites for microRNAs (miRNAs). They have been shown to be involved in different biological processes, and in particular, hematopoiesis [23], [24]. They are also described as behaving as tumor suppressor genes or oncogenes [25], [26]. Few new studies have reported on molecular abnormalities implicating miRNAs that can be up- or down-regulated in AML [27].

Table 8 and table 9 present the MCR from the data reported in the literature [18], [19], [20], [28], [29], [30], [31], [32], [33], [34]. Despite a certain amount of heterogeneity in the samples, the techniques, the genome constructs and other parameters, this table provides a global view of nearly 550 p-AML and 50 t-AML. It allowed us to clearly show the MCR and their frequencies.

**Table 8:** Minimal critical regions in the literature including the present work: losses.

Chromosome	All	p (%)	t (%)	consensus MCR	References	Genes
abnormalities				location in Mb		
1p36.2	6	6 (1)		7.81–7.89	A, D, E	PER3 UTS2
1p35.1	6	6 (1)		22.39–22.44	A, D, E	
1p34.2	3	3 (0.5)		42.07–42.45	A, D, E	KRC HIVEP3
1p31.3p31.1	3	2 (0.3)	1 (2)	67.07–69.95	A, D, E	WDR78 MIERS1 C1orf141 IL12RB2 SERBP1
						GADD45A GNG12 DIRAS3 GPR177 RPE65 DEPDC1
1q42.1	3	3 (0.5)		222.6–222.9	D	CNIH4 WDR26 CR625980 CNIH3
1q44	2		2 (4)	241.94–247.19	K	WDR64 EXO1 MAP1LC3C PLD5?CEP170 AKT3
						ZNF238 C1orf101 FAM36A EFCAB2 KIF26B
						SMYD3 TFB2M ADSS HNRNPU PPPDE1 AHCTF1
2p23.3	3	3 (0.5)		25.25–25.46	D, E	
2p23.1	3	3 (0.5)		30.29–30.95	A, D, E	
2q36.2	8	8 (1.4)		222.01–225.42	A, D, E	EPHA4 PAX3 FARSB MOGAT1 ACSL3 KCNE4 AP1S3
						WDFY1 SERPINE2 MRPL44 FAM124B CUL3 SCG2
3p26.3-p25.3	4	3 (0.5)	1 (2)	0–9.14	D, G	
3p26.3	4	3 (0.5)	1 (2)	14.96–16.15	A, D	
3p21.3	8	5 (0.9)	3 (6)	44.46–49.64	A, D, K	Numerous ZNF more than 30 genes
3p14.1p12.3	3		3 (6)	69.67–79.19	A, K	MITF FOXP1 EIF4E3 GRP27 PROK2 RYBP SHQ1
						PPP4R2 PDZRN3 CNTN3 FAM86D FRG2C ZNF717
						ROBO2 ROBO1
3q11.2q13.3	5	5 (0.9)		95.03–120.21	D, E	more than 30 genes
3q13.1q13.3	2	1 (0.2)	1 (2)	106.75–109.60	D	
3q13.3	3	2 (0.3)	1 (2)	117.78–118.40	D	
3q24q29	3	2 (0.3)	1 (2)	160.62–162.14	D	
3q24q29	3	2 (0.2)	1 (2)	164.51–165.39	D	SI SLITRK3 CR612557
4q24	7	7 (1.2)		106.1–106.72	A, D	TET2 PPA2 FLJ20184 INTS12 GSTCD
4q27q28	6	5 (0.9)	1 (2)	122.92–124.45	D	ADAD1 IL21 BBS12 FGF2 NUDT6 SPATA5 SPRY1
4q31.2	4		4 (8)	146.62–146.67	D, K	SMAD1
5q11.1	3	2 (0.3)	1 (2)	50.16–50.21	D	
5q11.2	3	3 (0.5)		51.79–53.66	D	
5q11.2	5	4 (0.7)	1 (2)	55.73–56.92	A, D	
5q11.2	4	3 (0.5)	1 (2)	57.49–57.79	A, D	
5q12.1	7	5 (0.9)	2 (4)	59.54–59.87	A, D, E	
5q13	14	10 (1.9)	4 (8)	82.72–84.65	A, D, E, J	VCAN HAPLN1 EDIL3
5q14.3q15	20	17 (3)	3 (6)	86.54–95.19	A, D, E, J	CCNH TMEM161B MEF2C CETN3 POLR3G
						RASA1 LYSMD3 GPR98 VLGR1 ARRDC3 NR2F1
						FAM172A POU5F2 ANKRD32 MCTP1 UNQ630
						TTC37 ARSK GPR150 RFESD RHOBTB3 GLRX
5q15q22.3	20	16 (2.6)	4 (8)	95.51–114.14	A, D, E, J	PCSK1 CAST ERAP1 ERAP2 LNPEP LIX1 2
						RIOK CHD1 PAM HISPPD1 NUDT12
						CR610784 RAB9P1 EFNA5 FBXL17 FER PJA2
						MAN2A1 TMEM232 TSLP WDR36 CAMK4
						STARD4 C5orf13 EPB41L4A APC SRP19
						ZRSR2 REEP5 DCP2 MCC YTHDC2 KCNN2
5q22.3q31.1	16	13 (2.3)	3 (6)	129.23–131.96	A, D, E, J	CHSY3 LYRM7 CDC42SE2 RAPGEF6 FNIP1 ACSL6
						CSF2 IL3 P4HA2 PDLIM4 IRF1 RAD50
5q31.1	21	12 (2.1)	9 (18)	133.52–134.26	A, D, E, H	PPP2CA CDKL3 UBE2B PHF15 SAR1B CAMLG
						DDX46 C5orf24 TXNDC15 PCBD2
5q31.3q33.1	19	11 (2)	8 (16)	142.91–148.19	A, D, E, J, K	HMHB1 YIPF5 PRELID2 GRXCR2 RBM27 POU4F3 LARS GPR151 PPP2R2B TCERG1 STK32A JAKMIP2
						SPINK1 SPINK5 SPINK6 SPINK7 SPINK9 FBXO38
						HTR4 SH3TC2
5q33.3	14	10 (1.8)	4 (8)	156.39–156.51	A, D, E, J	
5q33.3	13	10 (1.8)	3 (6)	157.25–158.80	A, D, E, J	CLINT1 EBF1 RNF145 UBLCP1 IL12B
6p25.1p24.3	10	7 (1.2)	3 (6)	5.54–8.38	A, B, C, D	FARS2 NRN1 F13A1 LY86 MMD-1 Zep-1
						RREB1 SSR1 CAGE1 RIOK1 DSP BMP6
						TXNDC5 MUTEDEEF1E1
6p22.3p22.2	5	4 (0.7)	1 (2)	20.29–23.74	A, B, D	
6p22.2p22.1	6	5 (0.9)	1 (2)	24.15–26.16	A, B, D	DCDC2 KAAG1 MRS2 GPLD1 ALDH5A1 TTRAP
						ACOT13 GMNN FAM65B DKFZp686H12134
						HIST1H cluster TRIM38 HFE
6q24.1	2	1 (0.2)	1 (2)	140.6–142.5	D, F	
6q25.3	2	1 (0.2)	1 (2)	156.39–156.61	D	
7p22.2	7	1 (0.2)	6 (12)	3.02–3.12	D, K	CARD11
7p14.1	4	3 (0.5)	1 (2)	39.5–41.7	D, F	
7p12.2	2	2 (0.3)		50.18–50.45	D	IKZF1
7q21.1	4	3 (0.5)	1 (2)	85.41–86.44	A, C, E	
7q21.1	5		5 (10)	90.21–90.44	A, D	PFTK1
7q21.3q22.1	14	6 (1)	8 (16)	97.69–100.36	A, D, E, K	LMTK2 BHLHA15 TECPR1 BRI3 NPTX2
						TMEM130 TRRAP SMURF1 KPNA7 MYH16
						ARPC1A PDAP1 BUD31 PTCD1 CPSF4 ATP5J2
						ZKSCAN5 CYP3A4 TRIM4 COPS6 MCM7
						TAF6 GATS PILRB PILRA ZCWPW1 MEPCE
						FBXO24 GNB2 EPO mir-25 mir-93 mir-106b
7q31.3	16	15 (2.7)	1 (2)	121.7–123.9	B, D, F, J, K	PTPRZ1 LKR/SDH AASS FEZF1 CADPS2 RNF133
						TAS2R16 NDUFA5 ASB15 WASL HYAL4
7q33q34	22	15 (2.7)	7 (14)	135.74–137.48	B, D, E, J, K	CALD1 TMEM140 STRA8 WDR91
						CNOT4 NUP205 FAM180A TPN miR490
7q34	20	19 (3.4)	1 (2)	139.72–140.02	B, D, E, J, K	TBXAS1 PARP12 MST109 JHDM1D KIAA1718
7q35q36.1	16	16 (2.9)		146.12–148.2	B, D, E, J, K	CNTNAP2
7q36.1q36.2	16	15 (2.7)	1 (2)	152.02–152.80	A, B, D, E, J,	MLL3 XRCC2 ACTR3B FABP5L3
					K	
8p23.2	3	2 (0.3)	1 (2)	3.48–3.58	A, D	
8p22p21.3	2	1 (0.2)	1 (2)	18.72–21.93	D	
8p21.2p12	2	1 (0.2)	1 (2)	24.40–36.30	D	
8q22.1	3	3 (0.5)		93.15–94.17	A, K	C8orf83
8q24.1	4	3 (0.5)	1 (2)	117.81–118.38	I	
8q24.1q24.2	4	3 (0.5)	1 (2)	126.54–129.58	A, D	
8q24.2	3	3 (0.5)		130.53–130.80	A, D	
8q24.2q24.3	3	2 (0.3)	1 (2)	131.51–146.26	A, D	
9p24.3p13.3	4	4 (0.7)		0.19–33.18	A, D, E	
9q21.3	5	4 (0.7)	1 (2)	82.37–84.44	B, C, D, E	
9q22.3q31.1	5	5 (0.9)		93.92–103.96	C, D, E, K	more than 30 genes
9q32q33.1	2	2 (0.3)		116.51–117.92	B, D	
10q23.1q23.3	4	4 (0.7)		87.93–90.77	C, D	
10q24.1q24.2	4	4 (0.7)		96.48–100.81	C, D	
11p15.5p15.4	8	7 (1.2)	1 (2)	2.48–3.35	B, C, D, E	KCNQ1 KCNQ1OT1 KCNQ1DN CDKN1C
						SLC22A18NAP1L4 PHLDA2 CARS
						OSBPL5 C11orf36 MRGPRE
11q12.3q13.1	3	3 (0.5)		63.98–65.65	D	
11q13.1q25	4	4 (0.7)		67.25–134.24	B, D, E	
11q23.3	4	2 (0.3)	2 (4)	117.86–119.81	A, D, E	
12p13.3	4	2 (0.3)	2 (4)	0.06–4.05	D, E	
12p13.2	10	6 (1)	4 (8)	10.62–11.72	C, D, E, J	
12p13.2	12	10 (1.8)	2 (4)	11.2–12.70	C, D, E, F, J	
12p13.2p13.1	17	14 (2.5)	3 (6)	12.20–13.13	C, D, E, J, K	ETV6 BCL2L14 LRP6 MANSC1 LOH12CR1
						AX748225 DUSP16 CREBL2 GPR19 CDKN1B
						APOLD1 DDX47 GPRC5A TVAS4 HEBP1
12p12.3	7	5 (0.9)	2 (4)	15.54–19.17	C, D, E, J, K	PTPRO EPS8 STRAP MGST1 LMO3 RERGL PIK3C2G
						CAPZA3
12q12	3	2 (0.3)	1 (2)	42.64–43.26	A, D	
12q13.1	2	2 (0.3)		46.06–46.30	A, D	
12q24.3	4	3 (0.5)	1 (2)	81.57–105.06	A, D, E	
12q24.3	3	2 (0.3)	1 (2)	121.28–122.73	A, D	
12q24.3	3	3 (0.5)		128.71–128.91	A, D, E	
13q14.3	8	7 (1.2)	1 (2)	49.63–50.51	C, D, E	
13q21.1	4	4 (0.7)		54.33–54.45	B, D	
16p11.1q12.1	20	20 (3.6)		35.06–50.12	D, G	VPS35 SHCBP1 ORC6L MYLK3 GPT2 DNAJA2
						NETO2 ITFG1 PHKB ABCC12 ABCC11 LONP2 SIAH1
						LONP N4BP1 CBLN1 HEATR3
16q21	24	23 (4.1)	1 (2)	57.56–58.96	B, D, G	GPR114 GPR56 GPR97 CCDC135 KIFC3 CNGB1
						TEPP MMP15 CSNK2A2 GINS3 NDRG4 SETD6
						CNOT1 GOT2
16q21	26	24 (4.3)	2 (4)	62.51–63.81	D, G, J	
16q22.3	25	22 (4)	3 (6)	70.76–71.46	D, G, J	VAC14 HYDIN FTSJD1 CALB2
16q22.3q23.1	26	26 (4.7)		73.30–73.57	G, H, J	BC042734
16q24.1	24	23 (4.1)	1 (2)	83.37–85.07	D, G, J	CDH13 HSBP1 MLYCD OSGIN1 NECAB2 MBTPS1
						HSDL1 LRRC50 TAF1C ADAD2 KCNG4 USP10
16q24.3	26	26 (4.7)		87.30–87.37	G, J, K	FBXO31
17p13.2p13.1	22	16 (2.9)	6 (12)	4.01–8.20	A, D, J	TP53 NLRP1 NUP88 C1QBP XAF1 and more than
						30 genes
17p12p11.2	7	5 (0.9)	2 (4)	14.74–19.35	A, D, E	
17p11.2	4	3 (0.5)	1 (2)	20.02–21.45	C, D, E	
17q11.2	44	39 (7.1)	5 (10)	26.49–27.52	A, D, E, G,	NF1 EVI2A EVI2A
					H, J, K	
17q21.33	4	2 (0.3)	2 (4)	44.98–45.43	D, E	
17q21.33	3	3 (0.5)		46.22–47.56	D, E	
17q25.1q25.3	2	2 (0.3)		69.43–73.16	D	
18p11.32p11.31	14	12 (2.1)	2 (4)	1–4.62	D, G, J	C18orf2 METTL4 NDC80 SMCHD1 EMILIN2
						LPIN2 MYOM1 MYL12B TGIF1 DLGAP1
18p11.23	17	16 (2.9)	1 (2)	7.42–7.58	G, H, J	PTPRM
18p11.22p11.21	5	4 (0.7)	1 (2)	9.03–13.81	D, H	
18q11.2	9	8 (1.4)	1 (2)	18.1–22.05	D, G	
18q11.2q12.2	21	18 (3.2)	3 (6)	21.74–33.13	D, G, K	CABYR OSBPL1A IMPACT HRH4 ZNF521 SS18
						PSMA8 TAF4B CDH2 DSC3 DSC1 DSC2 DSG4
						DSG3 DSG2 TTR RNF125 DTNA MAPRE2
18q21.1	21	20 (3.6)	1 (2)	43.39–46.37	D, E, G, K	HAUS1 LOXHD1 PIAS2 SMAD2
18q21.2	22	20 (3.6)	2 (4)	50.21–51.05	D, E, G, K	RAB27B CCDC68
18q21.32	22	20 (3.6)	2 (4)	55.08–55.51	D, E, G, J, K	ONECUT2 FECH NARS ATP8B1
18q22.3	23	22 (4.0)	1 (2)	69.06–69.18	D, E, G, K	
18q22.3q23	4	2 (0.3)	2 (4)	71.06–71.76	E, G, J	FBXO15
19p13.3	3	2 (0.3)	1 (2)	0.21–1.81	A, D	
19q13.2q13.43	2	2 (0.3)		46.16–63.43	C, D	
20q11.2q13.1	2	2 (0.3)		34.59–43.45	E	more than 30 genes
20q13.1q13.3	2	2 (0.3)		43.85–55.73	E	more than 30 genes
21q22.12	11	7 (1.2)	4 (8)	35.11–35.15	A, D, K	RUNX1
21q22.3	3	3 (0.5)		44.70–45.09	D	

The first column lists the chromosomal losses and gains. The second lists the absolute number of each rearrangement (excluding a single rearrangement). The third and the fourth are the absolute numbers in p-AML and t-AML respectively and the percentage is indicated between parenthesis. The chromosomal location is listed. The references are indicated by letters: A [32], B [33], C [34], D [18], E [19], F [28], G [29], H [30], I [31], J [20], K present work. The last column lists the genes included in those MCR.

**Table 9:** Minimal critical regions in the literature including the present work: gains.

Chromosome	All	p (%)	t (%)	consensus MCR	References	Genes
abnormalities				location in Mb		
GAINS						
1q21.3q22	4	3 (0.5)	1 (2)	153.19–154.64	C, D, K	S100A8 S100A9 S100A13 S100A6 SNAPIN ILF2
						NPR1 INTS3 VLCS-3 GATAD2B TORC2 CREB3L4
						TPM3 HAX1 IL6R SHE UBE2Q1 ADAR CHRNB2
1q32.1	3	2 (0.3)	1 (2)	204.98–205.04	C, D	
1q32.1q44	6	5 (0.9)	1 (2.1)	235.00–235.10	A, B, K	BC016972 ?
3q26.3q29	2	2 (0.3)		182.99–133.31	E	more than 30 genes
4p13q12	2	2 (0.3)		42.30–58.23	B, D	
4q12q13.1	2	2 (0.3)		59.13–62.98	B, D	
4q28.3	2	2 (0.3)		132.8–136.7	B, G	
5p13.2q11.1	2	1 (0.2)	1 (2)	37.98–50.15	A, D	
5q11.2	3	2 (0.3)	1 (2)	51.21–54.12	A, D	
6q11.2q12	8	8 (1.4)		63.43–63.7	A, G	
6q27	3	2 (0.3)	1 (2)	168.12–168.32	D, K	MLLT4 avoir table 6
7p15.2	4		4 (8)	27.15–27.18	K	HOX genes
8q24.3	16	12 (2.1)	4 (8)	143.73–143.90	B, D, G, I, J, K	JRK PSCA LY6K C8orf55 LYPD2 SLURP1 LYNX1
						LY6D
9p21.3	2	2 (0.3)		21.5–23.4	F, G	
9q13q21.3	2	2 (0.3)		70.23–83.52	E	FXN APBA1 TJP2 SMC5 TRPM3 GDA TCM1 ANXA1
						RARB RFK GNA14 TLE4 TLE1
11q12.1q14.1	16	16 (2.9)		59.1–79.6	G	more than 50 genes
11q14.1	5	5 (0.9)		79.6–81.3	D, G	
11q24.2q24.3	31	31 (5.6)		126.53–130.23	D, E, G, J	KIRREL3 ETS1 FLI1 C11orf45 TP53AIP1 RICS
						BARX2 NFRKB PRDM10 APLP2 ST14 ZBTB44
12p13.33p11.21	13	13 (2.3)		0.1–32.7	E, G	no gene
13q12.11q13.1	3	3 (0.5)		19.9–31.7	G	
13q31.3	2	2 (0.3)		90.45–90.97	E	GPC5
15q21.1	3	3 (0.5)		46.52–47.36	B, D, E	
15q21.3q22.1	3	3 (0.5)		55.6–56.6	B, E, F	
15q23	5	5 (0.9)		67.44–68.37	B, E, J	SMAD3 AAGAB MAP2K5 LBXCOR1 PIAS1
15q26.q26.3	6	6 (1)		92.5–100.0	C, E, G	
17q12	6	4 (0.7)	2 (4)	32.82–33.20	C, D, E, J	TMEM132E
17q21.2q21.31	5	3 (0.5)	2 (4)	37.08–38.48	D, E, J	FBXO47 PLXDC1 FBXL20 MED1 CRKRS TCAP
						PGAP3 ERBB2 GRB7 IKZF3 GSDMB ORMDL3
						PSMD3 CSF3 MED24 THRAP4 NR1D1 MSL1 CASC3
						WIPF2 CDC6 RARA
19p13.3p13.12	13	13 (2.3)		2.19–14.11	D, G, J	
19p13.12p13.11	13	13 (2.3)		16.0–16.15	F, G, J	
19q13.31	2	2 (0.3)		49.2–49.9	D, F	
19q13.41	2	2 (0.3)		58.3–59.1	D, G	
20q11.1q11.21	3	3 (0.5)		28.2–30.5	E, G	
21q21.1	2	2 (0.3)		14.29–17.97	K	LIPI ABCC13 RBM11 HSPA13 SAMSN1 NRIP1
						USP25 C21orf34 CXADR BTG3
						miR-99a miR-125b-2 hsa-let-7c
21q22.2	18	18 (3.2)		38.65–38.85	C, D, G, J, K	ERG ETS2
21q22.3q	4	4 (0.7)		45.09–46.91	D, K	RRP1B PDXK RRP1 CSTB AGPAT3 TRAPPC10
						TMEM1 PWP2 C21orf33 ICOSLG DNMT3L AIRE
						PFKL TRPM2 LRRC3 UBE2G2 SUMO3 PTTG1IP PBF
						ITGB2 ADARB1 COL18A1
22q12.3	13	12 (2.1)	1 (2)	33.90–35.32	D, G	LARGE
22q13.1q13.2	14	13 (2.3)	1 (2)	36.78–42.24	D, G	more than 30 genes
22q13.31q13.32	11	10 (1.8)	1 (2)	44.92–48.08	D, G	PRR5 ARHGAP8 PHF21B NUP50 UPK3A SMC1B
						ATXN10 WNT7B GTSE1 GRAMD4

The first column lists the chromosomal losses and gains. The second lists the absolute number of each rearrangement (excluding a single rearrangement). The third and the fourth are the absolute numbers in p-AML and t-AML respectively and the percentage is indicated between parenthesis. The chromosomal location is listed. The references are indicated by letters: A [32], B [33], C [34], D [18], E [19], F [28], G [29], H [30], I [31], J [20], K present work. The last column lists the genes included in those MCR.

When we compared the number of losses and gain in the “table” sample to those in the present series, a chi2 test showed that as they were not different, we could include them in the table. In all AML 123 MCR were losses and 51 were gains. In t-AML, 34 MCR were losses and 4 were gains while in p-AML 34 were losses and were 4 gains.

We will examine the lost and gained MCR found in our series together with the most specific or frequent MCR found in this table.

### −1q

A small juxta-telomeric MCR loss in -1q44 was not reported in p-AML and does not seem to be very frequent (tables 8 and 9). Among more than twenty genes, SMYD3 is a histone methyltransferase that plays a role in transcriptional regulation as a member of an RNA polymerase complex. It is expressed in CD34 cells.

### −3p

Two MCR were found. The most telomeric one on 3p21.3 was a 5.18 Mb recurrent loss covering more than 40 genes. It was found in p-AML (table 6, figure 1) but also in t-AML (tables 8 and 9).

A more centromeric 10Mb loss was found exclusively in t-AML. Both patients in the present work contributing to this MCR had been treated with RT and 5FU. PROK2, a prokineticin 2 isoform A precursor, is highly expressed in bone marrow [35] and could have chemokine-like activity [36]. Another gene, the SHQ1 homolog, expressed in CD34 cells has been purported to be required for the assembly of H/ACA small nucleolar and telomerase RNPs [35], [37].

### −4q

A specific loss in p-AML was found in 4q24 (table 8) including TET2, a tumor suppressor gene described in myeloid cancers [38].

A small loss of 4q31.2, exclusively in 8% of t-AML (tables 7 and 8) was found to delete the 5′ half of SMAD1. This protein mediates the signals of bone morphogenetic proteins (BMPs), which are involved in a range of biological activities including cell growth, apoptosis, morphogenesis, development and immune responses. It is expressed in BM cells (UCSC).

### −5q

The 5q- region was fragmented in more than twelve groups. In p-AML, the most frequent regions were comprised between 86.54 and 114.14 Mb, with proximal and distal “spreading”. This region contains the RASA1 gene which exhibits tumor suppressor activity on the RAS gene. RASA1 has been reported to be a CNA in breast cancers and a putative tumor suppressor gene [39]. PCSK1 may be implicated in malignancy [40]. APC and MCC genes are implicated in colorectal cancer. The more proximal area contains the AML/MDS region that Evers considered as a 5.2 Mb MCR [41].

Two MCR were observed in more than 10% of t-AML (table 8). Both were proximal to the previously reported 7.7 Mb 5q33.3 region [41]. The first MCR was located on 5q31 and was smaller than 1Mb. It contained several genes of interest such as CDKL3, an important regulator of cell cycle progression, UBE2B that is required for post-replicative DNA damage repair, and the helicase DDX46 which is highly transcribed in bone marrow.

The second 5.22 Mb MCR in 5q31.3q32, was delineated by the present work (table 6). Among the 29 genes it harbored, several are overexpressed in bone [35]. PRELID2 codes for a protein containing a PRELI/MSF domain. It is an evolutionary conserved gene [42], [43]. TCERG1 is a transcription factor that binds RNA polymerase II, inhibits the elongation of transcripts from target promoters and regulates transcription elongation in a TATA box-dependent manner. LARS encodes a cytosolic leucine-tRNA synthetase, a member of the class I aminoacyl-tRNA synthetase family. FBXO38 is a F-box protein 38 isoform A that is overexpressed in early erythroid cells. FBXO32, a family member of these cells, is a PRC2-targeted gene [44]. POU4F3, a POU class 4 homeobox 3 is a member of the POU-domain family of transcription factors that is not expressed in bone marrow but in monocytes. Furthermore, another member of the POU gene family, POU4F1, has been recently described to be associated with AML exhibiting t(8;21) [45]. RBM27 is a zinc finger RNA-binding protein member of the family that includes RBM15, alias OTT, which is involved in the regulation of hematopoietic stem cells and is fused with MKL1 in the t(1;22) of AML7 and plays a major role in the pathogenesis of this disease [46].

Finding different MCR with a maximum frequency in p-AML and in t-AML suggests different oncogenesis pathways on this chromosome.

### 7p MCR

A 90kb MCR in 7p22.2 (table 6 and 8) had deleted he CARD 11 gene in t-AML. The CARD domains of this caspase have been shown to activate NF-kappaB and to induce the phosphorylation of BCL10 when expressed in CD34+ cells [47]. CARD 11, via the immune cell restricted complex CARD11-BCL10-MALT1, [48] is implicated in lymphoma.

In three t-AML cases, a gain of 7p15 concerned the homeogene cluster of HOXA6, HOXA7, HOXA9 and HOXA10 (Table 6). This area has not been described as a CNV [7]. The HOXA family of genes encode HOX family transcription factors, which play an important role in the development of body segmentation and in the survival of hematopoietic stem cells. HOXA6 is directly implicated in the process of hematopoietic progenitor cell development. HOXA9, that can be fused with NUP98 in some AML [49], is fundamentally involved in the AML process in transgenic mice [50], [51], [52].

These genes belong to the HOXA5-11 cluster and are expressed throughout the CD34+ compartment [53], [54]. The CD34+ level becomes important in AML due to the immature status of blastic cells.

As overexpression of Abd-B HOXA genes has been demonstrated in AML with a rearranged MLL [55], the gain of the HOXA cluster could be expected in case t-19 which had a t(9;11). The other t(v;MLL) did not exhibit this gain. The other two patients with such a gain (cases t-17 and t-14) had a t(15;17) and a +8 respectively. They were cytologically and cytogenetically different and had wild type MLL. The HOX gain was not detected in p-AML.

### −7q

On 7q, the 11 MCR with a specificity for t-AML or p-AML were interlaced. Three MCR were reported in more than 10% of t-AML (table 8). The first one is 7q21.1 with a small region potentially implicating PFTK1, a member of a protein kinase family whose gain was recently shown to be involved in hepatocellular carcinoma cells.

The most frequent MCR in t-AML was 7q21.3q22.1 (tables 6 and 8). The number of genes present in this almost 3 Mb-long MCR prohibited extensive reviewing.

Three miRNA, mir-25, mir-93 and mir-106b were present in this deleted region (table 6 and table 8), regulating numerous genes. The miR-106b∼25 cluster cooperates with its host gene MCM7 in cellular transformation both *in vitro* and *in vivo*, so that the concomitant overexpression of MCM7 and the miRNA cluster triggers prostatic intraepithelial neoplasia in transgenic mice [56]. MCM7 can be associated with CDK4 that may regulate the binding of this protein to the tumor suppressor protein RB1/RB. Among 30 genes, BRI3 appears to be overexpressed in BM-CD33+ cells. It seems to play a key role in TNF-induced cell death [57].

A defect in DNA repair was reported in TRRAP-deficient cells [58].

In 7q33q34, a 1.74Mb MCR had a low ratio in a patient (t-2), implying a probable double deletion, while the remaining ratio could have been due to the few normal cells. This MCR was the third in t-AML. It contained miRNA-490 which was probably regulating fewer than a hundred genes, with a maximum context score for the FOS gene. Among several genes (table 6), CREB3L2 was overexpressed in BM-CD34+ cells. It is a DNA binding and basic leucine zipper dimerization (bZIP) transcription factor. It was reportedly fused with FUS in fibrosarcomas [59] via a t(7;16)(q34;p11) and with PPAR gamma in a subset of thyroid carcinoma via a t(3;7)(p25;q34) [60].

In our series of p-AML, a large 43 Mb MCR was situated between 7q31.3 and the telomere (table 6). Table 8 allowed us to divide this region into 5 smaller MCR that seemed to be different from those of t-AML. The only exception was the previously identified 7q33q34 MCR that was the only MCR common to t-AML and p-AML in the loss of 7q.

The most centromeric p-AML-specific deletion was a 2.2Mb MCR on 7q31.3. Among the 10 genes in the region, only WASL is reported to potentially play a role in the microthrombocytopenia, the characteristic sign of Wiskott-Aldrich syndrome [61].

The 7q34 MCR was the most frequent and the most specific in p-AML. It contains 5 genes (table 8) that are not obviously involved in malignancy and some (e.g. PARP12) are poorly known but all are more or less strongly expressed in CD33 myeloid cells.

They were located between 7q31.3 and 7q36.2. Obviously, there are too many genes in those 5 MCRs, but PTPRZ1, MLL3 and XRCC2 could be candidate leukemogenic genes.

### +8q24.3

A gain of 170 Kb located on 8q24.3 was selected as a MCR from two cases in the present group of t-AML (Figure 1). This MCR was confirmed in other series (table 9), frequently in t-AML (8%) and less frequently in p-AML (2.1%). This finding is consistent with the +8q22qter MCR being the “drive” genes of trisomy 8 which can be observed in some t(8;21) AML [62]. This MCR was telomeric to the MYC and TRIB1 genes [63]. PSCA, implicated in prostate cancer and C8orf55, the “mesenchymal stem cell protein DSCD75” which plays a role in bone marrow cell interactions, could not be excluded. However JRK, which is moderately overexpressed in BM-CD34+ cells [6] encodes a putative DNA-binding protein [64] and exhibits homology with the CENP-B (centromere-binding protein B).

### +11q24.2q24.3

Although no such result was found in our study, 5.6% of the p-AML in other series clearly showed gains or amplifications of this region that contains ETS1 and FLI1 genes. They are members of the ETS gene family that includes genes playing important roles in regulating hematopoiesis, proliferation, differentiation and apoptosis. Interestingly, these gains were found in cases presenting gains of the ETS2 and ERG genes (see below, the +21q22 paragraph).

### −12p13

A 1.48Mb loss was defined as a MCR from three t-AML cases in the present report. The same area is recurrently lost in both types of AML (table 7, table 8). Among 17 genes, the 5′ part of ETV6 was lost. This ETS family transcription factor is a multi-partner gene with almost 28 different fusion genes via reciprocal translocations. It is implicated in multiple myeloid malignancies such as MDS and AML, but also in lymphoid malignancies and in fibrosarcomas [49]. CDKN1B encodes a cyclin-dependent kinase inhibitor. These two genes are considered as having tumor suppressor activity.

### −17p13.2p13.1

Although no deletion of this region was found in our series, several reports claimed a loss of this MCR in 12% of t-AML and 3% of p-AML. This region, among several genes, exhibits loss of the TP53 tumor suppressor gene [49].

### −17q11.2

This MCR, that we found in a single case of t-AML (table 7), is the most frequent loss of the table 8 with 7.1% of in p-AML and 10% of in t-AML. This MCR contains the NF1 tumour suppressor gene NF1 which encodes neurofibromin, a GTPase-activating protein that negatively regulates RAS signaling by stimulating hydrolysis of Ras-GTP. Loss of NF1 can lead to a progressive myeloproliferative disorder in animal models [65] and in Juvenile myelomonocytic leukemia. Parkin [19] concluded that NF1 null states were present in 7% of AML. This important point confirmed that additional events were are required.

### −18

Loss of different parts of chromosome 18 were highly recurrent in p-AML (table 8). In our series, we found a single p-AML with a 2.06 Mb deletion that had an equivalent MCR in table 8 which contains RAB27B and CCDC68 genes.

### −21q22.12

This 40kb small MCR contains RUNX1 that was lost in 4 t-AML cases in the present series. Two patients had lost their two alleles either through double losses or deletion and D171N mutation. This mutation has been claimed to result in the proliferation of immature myeloid cells, an enhanced capacity for self-renewal, and the proliferation of primitive progenitors [66].

Patient t-29 had a normal karyotype [32] and a 310kb deletion that deleted most of the gene in a radiotherapy-induced leukemia [67].

**Figure 2:**
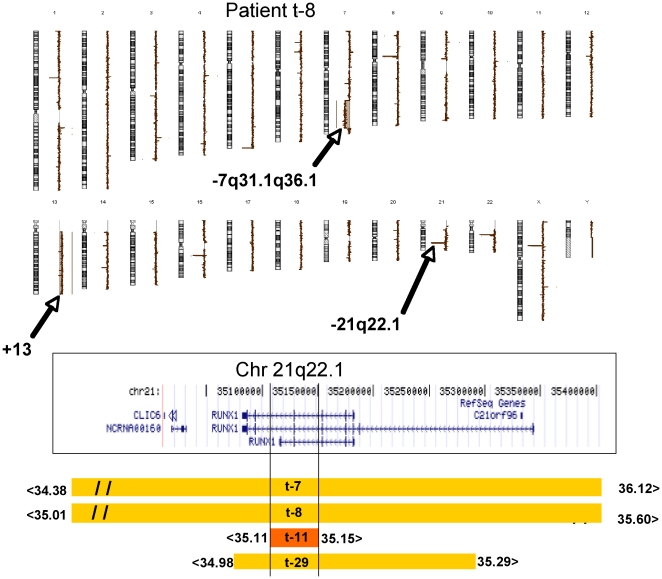
aCGH karyogram of patient t-8 and MCR delineation of RUNX1. A = A del (7q) and trisomy 13 are obvious. Cryptic deletions from 21q22.1 (corresponding to RUNX1) and from Xp11.4, that are smaller than 60kb, are nullosomic. On 22q11, the loss is a constitutive CNV of GGT1. B = A UCSC map (build 18) of the RUNX1 gene. C = The deletions of RUNX in four patients at the molecular level are labeled in orange and the smallest in red. The location of the breakpoints are indicated at the ends of the colored lines. Patient t-8 exhibits a homozygous 590 Kb deletion that encompassed the entire RUNX1 gene and could have occurred by an acquired isodisomy. Patient t-11 had the smallest deletion (40Kb) that was internal to RUNX1; t-29 had a deletion limited to RUNX1.

Three deleted cases were exposed to multi-agent chemotherapy with alkylating agents. They exhibited a 7q-/-7 as previously reported [68] as well as patient t-12 that had a balanced abnormality involving RUNX1, only detected by classical karyotype. It has been suggested that the mutation of RUNX1 and gene losses localized on 7q could cooperate in leukemogenesis and predispose to leukemic transformation into t-AML following alkylating agents (figure 2). In table 8, seven other cases were observed in p-AML.

RUNX1, the subunit alpha of the Core Binding Factor (CBF), a heterodimeric transcription factor, plays a key role in the regulation of hematopoietic stem cell proliferation and differentiation. It is one of the most frequent targets of chromosome translocations via nearly forty different partners [49] in various forms of leukemia.

A germline mono-allelic mutation or deletion of RUNX1 [69] has been described in FPD (Familial Platelet Dysfunction) disease. A second mutation may appear at the AML stage, mostly with an M0 type in the FAB classification [70].

Point mutations of RUNXI have been described in radiation-induced MDS [67] or in therapy-induced transformation of myeloproliferative neoplasia [66].

Haploinsufficiency of RUNX1 leads to the loss of function of this gene (probably a tumor suppressor function). In the case of t-AML, the various point mutations are localized at the N-terminal, the DNA binding site [68].

### +21q22

Distal 21q11.2qter polysomy was observed in three cases (table 6), two of which exhibited amplifications of this region in the context of a complex karyotype [21].

The first amplicon (table 5 and 6) from 14.29 to 17.97Mb harbored a cluster of miR-99a, miR-125b-2 and hsa-let-7c, each of which is predicted to regulate several hundred genes. Several of the genes in this amplicon (table 6) were overexpressed [35] in BM-CD34+ cells. SAMSN1, a hematopoietic adaptor was found to contain the SH3 and SAM domains 1, HSP13, BTG3 and NRIP1. This region was reported to be amplified by BAC aCGH in a series of patients with complex karyotypes [71] which showed that NRIP1 and SAMSN1 genes were up-regulated compared with their status in patients with normal karyotypes. The same area from LIP1 to miR-125b-2 is reported to be homozygously deleted in the Non-Small Cell Lung Cancer cell line Calu-6 [72].

The 1Mb-long second amplicon, observed in cases p-21 and p-24 was found to occur frequently in p-AML (table 9) and most of the cases also exhibited a gain of ETS1. It contained ERG and ETS2 genes. These two ETS transcription factor gene family members were overexpressed in BM-CD34+ cells. ERG can be fused with several genes in prostate cancer [73], in Ewing tumors [74] and in leukemias with a t(16;21)(p11;q22) and an FUS-ERG fusion [49]. It has been claimed that ERG is a megakaryocytic oncogene [75] together with ETS2 [76].

**Figure 3:**
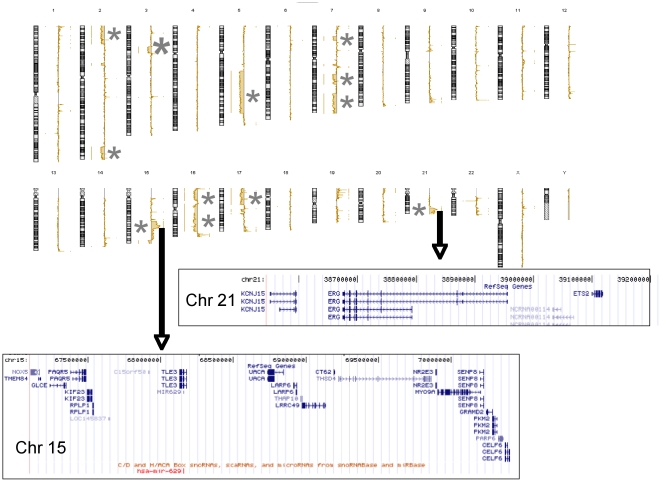
p-AML (case p24) with a complex karyotype. See the amplification of 15q23q24 and of 21q11.2q22.1 that are enlarged at the gene level. Multiple abnormalities (cf table 5) are asterisked.

ERG and ETS2 were amplified in p-AML with complex karyotypes (figure 3) and ETS2 overexpression was highly correlated with the amplification, unlike ERG [71], [77]. The preliminary results of a transcriptome study of case p-21 are in agreement with these findings. The RUNX1 region was not amplified in p-AML in our small sample.

### Conclusion

High resolution cytogenomics obtained by aCGH and similar techniques already published allowed us to characterize numerous untargeted non random chromosome abnormalities. This work supports the hypothesis that they can be classified into several categories: abnormalities common to all AML (e.g. 8q24.3 gain or 17q11.2 deletion involving NF1); those more frequently found in t-AML (e.g. 7q21 or 7q33 deletions or even the specific gain of HOX genes); and those specifically found in p-AML (e.g. loss of the 139 to 152.8 Mb of 7q, 11q24 gain or 21q22 with amplifications of ERG and ETS2).

The genes involved in AML MCRs are often very well known in leukemogenesis but many others need to be explored.
